# Synergistic Antioxidant and Anti-Ferroptosis Therapy via BPNS-Encapsulated Thermoresponsive Chitosan Hydrogel for Spinal Cord Injury Regeneration

**DOI:** 10.3390/pharmaceutics17050573

**Published:** 2025-04-26

**Authors:** Yang Liu, Yingkai Wang, Xiangzi Wang, Wanchen Zeng, Zehong Zhang, Zhengmian Zhang, Zhongquan Qi

**Affiliations:** 1 School of Medicine, Guangxi University, Nanning 530004, China; ly2228301010@163.com (Y.L.); wykay23@163.com (Y.W.); 3018001682@tju.edu.cn (X.W.); zzz22qc@163.com (W.Z.); 3150104352@zju.edu.cn (Z.Z.); 2 Fujian Maternity and Child Health Hospital, Fuzhou 350001, China; 3 Fujian Provincial Human Sperm Bank, Fuzhou 350001, China; 4 College of Clinical Medicine for Obstetrics & Gynecology and Pediatrics, Fujian Medical University, Fuzhou 350122, China; 5 Stem Cell Therapy Research Center, Fuzhou 350001, China

**Keywords:** spinal cord injury, black phosphorus nanosheets, hydrogel, reactive oxygen species, ferroptosis, apoptosis

## Abstract

**Background**: Spinal cord injury (SCI) is a devastating neurological condition with limited therapeutic options. Current clinical interventions predominantly rely on prolonged or high-dose pharmacological regimens, often causing systemic toxicity and adverse events. Although black phosphorus nanosheets (BPNSs) exhibit remarkable reactive oxygen species (ROS)-scavenging capacity to mitigate oxidative damage, their rapid degradation severely compromises their therapeutic efficacy. **Methods**: This study presents a thermosensitive hydrogel with rapid gelation properties by incorporating different proportions and concentrations of sodium alginate (SA) into a chitosan/β-glycerophosphate (CS/β-GP) hydrogel and loading it with BPNS for the treatment of SCI in rats. In vitro, the physical properties of the composite were characterized and the cytotoxicity and ROS scavenging abilities were assessed using PC12 cells; in vivo, behavioral tests, histopathological analysis, transcriptomics, immunohistochemistry, and Western blotting were performed to explore the therapeutic effects and mechanisms. **Results**: The results demonstrate that this hydrogel effectively slows BPNS degradation, exhibits a high ROS scavenging capacity, reduces lipid peroxidation, and thereby inhibits ferroptosis and apoptosis, offering neuroprotective effects and promoting motor function recovery. **Conclusions**: Our findings establish the CS/β-GP/SA-BPNS hydrogel as a multifunctional therapeutic platform for SCI, synergizing sustained drug release with ROS–ferroptosis–apoptosis axis modulation to achieve neuroprotection and functional restoration. This strategy provides a translatable paradigm for combining nanotechnology and biomaterial engineering in neural repair.

## 1. Introduction

Spinal cord injury (SCI) is a severe neurological injury that has long-term adverse effects on patients, their families, and society as a whole [1,2]. SCI can lead to serious sensory, motor, autonomic dysfunctions, and abnormal reflexes [3]. From the perspective of pathophysiology, it can be divided into three stages: acute phase, subacute phase, and chronic phase. It involves a series of complex and adverse processes such as oxidative stress, inflammatory response, ferroptosis, and apoptosis [4,5,6]. Currently, commonly used medications often rely on glucocorticoids and other drugs administered at high doses or for prolonged periods, which frequently lead to adverse effects [7]. Therefore, there is an urgent need for a safe and effective low-dose treatment approach.

During spinal cord injury, factors such as ischemia, hypoxia, and inflammatory responses intertwine, leading to the massive production of reactive oxygen species (ROS). This excess of ROS disrupts the intracellular redox balance, triggering oxidative stress [8,9]. As a critical factor, oxidative stress significantly impacts neuronal death after spinal cord injury [10]. Recent studies have revealed intricate connections between ferroptosis and the pathophysiological processes of spinal cord injury [11]. Ferroptosis, a type of cell death dependent on iron ions, plays a crucial role in this complex pathophysiological mechanism [12,13]. Related research indicates that oxidative stress is a key pathway triggering ferroptosis, wherein ROS react with polyunsaturated fatty acids (PUFAs), and the resulting lipid peroxidation is a critical step in the ferroptosis process [14,15]. Concurrently, during ferroptosis, the decomposition of lipid peroxidation products generates new ROS, further exacerbating oxidative stress and forming a vicious cycle [16,17]. Based on this, exploring methods to inhibit oxidative stress and ferroptosis holds promise for alleviating neuronal damage and motor dysfunction caused by spinal cord injury, offering new hope for spinal cord injury treatment. Additionally, the accumulation of ROS can induce apoptosis [18]. Apoptosis is a programmed cell death process mediated by caspase-related enzymatic reactions in damaged nerve cells after spinal cord injury, leading to the cleavage of structural and functional proteins within the cell [19]. This process involves the activation of a series of genes and the regulation of signaling pathways [20]. Among them, the interaction between the anti-apoptotic protein Bcl-2 and the pro-apoptotic protein Bax regulates cell death [21]. This process reduces the number of neurons, further hindering the recovery of neural function [22]. Therefore, inhibiting apoptosis can serve as a therapeutic direction for neuronal protection.

Black phosphorus (BP) is a layered material formed by stacking phosphorus layers with weak van der Waals forces, and black phosphorus nanosheets (BPNSs) are two-dimensional nanomaterials obtained by exfoliating black phosphorus [23]. BPNS degrades in vivo into non-toxic phosphates, phosphites, offering good biocompatibility [24,25]. Each phosphorus atom contains a lone electron pair, which can react with radicals in reactive oxygen species (ROS), such as superoxide and hydroxyl radicals, reducing the accumulation of ROS [26]. Studies have shown that BPNS effectively reduces ROS levels in models of osteoarthritis and kidney injury in rats [24,27]. Due to the presence of lone electron pairs in each phosphorus atom in BPNS, it easily reacts with oxygen and water in the environment, leading to uncontrolled degradation [28]. Therefore, a drug delivery method that reduces BPNS degradation rate and enhances its stability is needed.

Chitosan (CS), a natural polysaccharide derived from the deacetylation of chitin in nature and renowned for its excellent biocompatibility, degradability, and accessibility, has found extensive applications in biomedicine as a carrier for drug delivery [29]. When combined with the organic phosphate β-glycerophosphate (β-GP), CS forms a sol state at relatively low temperatures due to weakened intermolecular electrostatic repulsion and hydrogen bonding [30,31,32]. As the temperature approaches human body temperature, the sol gradually transitions into a gel, resulting in a thermosensitive hydrogel. Sodium alginate (SA), a natural biomaterial extracted from brown algae, has been shown to alter the gelation time of chitosan hydrogels [33,34]. Therefore, we added SA in various concentrations and ratios to the chitosan hydrogel to alter the gelation time and strength of the hydrogel, thereby obtaining an injectable, fast-gelling, biocompatible, and sustained-release thermosensitive hydrogel.

Research has found that loading BPNS with chitosan thermosensitive hydrogels effectively slows down BPNS degradation [35,36]. The instability of BPNS under physiological conditions, caused by rapid oxidation and uncontrolled degradation, restricts its biological availability. Hydrogels loaded with BPNS not only shield the nanosheets from environmental oxygen and moisture—thereby decelerating their degradation rate—but also leverage their thermosensitive characteristics to enable rapid in situ gelation following injection. This dual functionality guarantees the long-term retention of BPNS at the injury site while maintaining its antioxidant activity.

Current treatment strategies for SCI primarily focus on the systemic administration of high-dose drugs or surgical intervention, suffering from systemic side effects and inadequate drug accumulation at the injury site due to the blood–spinal cord barrier [37]. In contrast, BPNS enables local delivery through injectable hydrogels, achieving high drug concentrations at the injury site. Furthermore, the biodegradability of both BPNS and the hydrogel eliminates the need for secondary surgery to remove implants, addressing a major limitation of non-degradable implants [38]. And the injectable hydrogel provides a minimally invasive approach that reduces surgical trauma and infection risk.

**Scheme 1:**
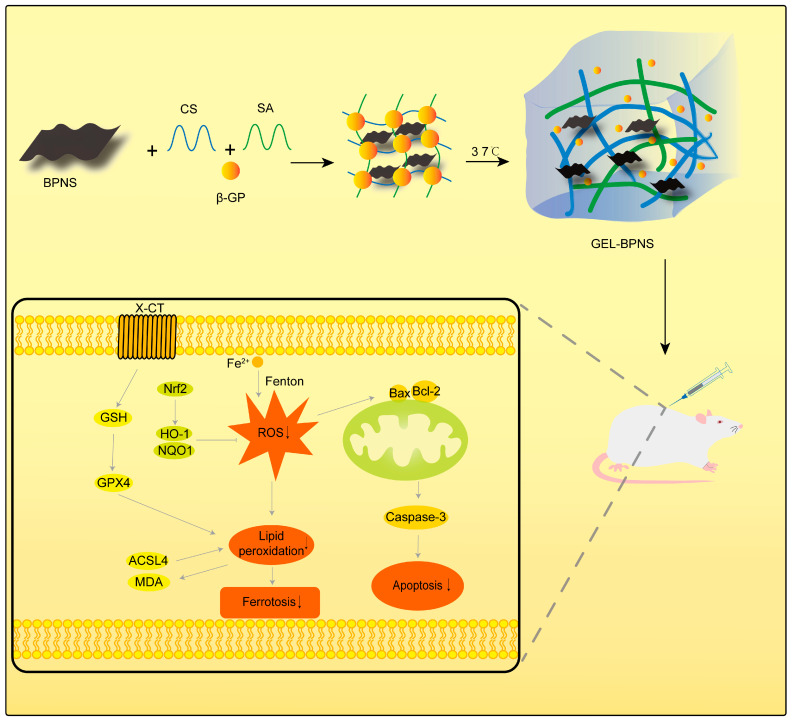
Schematic overview of the development of a thermosensitive hydrogel loaded with BPNS for SCI.

In this study, we developed a fast-gelling thermosensitive hydrogel system loaded with BPNS. The BPNS-loaded hydrogel was injected into the SCI site, and due to the rat’s body temperature, it rapidly gelled at the injury site, achieving the in situ sustained release of BPNS. We conducted in vitro and in vivo studies to examine the basic characteristics of the composite and its therapeutic effects on SCI in rats, focusing on exploring the mechanisms by which GEL-BPNS affects oxidative stress, ferroptosis, and apoptosis (Scheme 1). From a translational perspective, both CS and SA are FDA-approved materials with established safety profiles in biomedical applications [39], and the hydrogel preparation process is scalable under mild conditions. Furthermore, the injectable nature of GEL-BPNS aligns with the minimally invasive clinical requirements, offering a feasible strategy for future industrial production and commercialization.

## 2. Materials and Methods

### 2.1. Material Acquisition

Black phosphorus nanosheets (BPNSs, 0.1 mg/mL, average lateral size 200 nm, thickness 3–5 nm, prepared via liquid exfoliation) were obtained from Six Carbon Technology Shenzhen (Shenzhen, China). Chitosan (CS, degree of deacetylation ≥ 95%, CAS 9012-76-4), β-glycerophosphate disodium salt (β-GP, ≥95% purity, 13408-09-8), sodium alginate (SA, AR, low viscosity, 150–250 mPa·s, guluronic/mannuronic acid ratio 1.2, CAS 9005-38-3), and glacial acetic acid (≥99.7% purity, CAS 64-19-7) were purchased from Aladdin Chemistry Co., Ltd. (Shanghai, China). The culture medium used in cell experiments includes high glucose DMEM (Gibco, Waltham, MA, USA), 10% fetal bovine serum (Gibco, Waltham, MA, USA), and 1% penicillin/streptomycin (Gibco, Waltham, MA, USA).

### 2.2. Preparation of Hydrogels

Based on previous literature studies, we adopted the following methods to prepare the hydrogel [30,31,32,33]. We dissolved 250 mg of chitosan into 11.25 mL of 0.1 mol/L acetic acid solution and stirred at room temperature for 6 h to prepare a CS solution, which was then stored at 4 °C. We dissolved 700 mg of β-glycerophosphate sodium into 1.25 mL of deionized water to prepare a β-GP aqueous solution, which was also stored at 4 °C. We dissolved 30 mg/60 mg/120 mg of sodium alginate into 10 mL of deionized water separately, and stirred at room temperature for 2 h until completely dissolved to prepare SA solutions with mass fractions of 0.3%, 0.6%, and 1.2%.

Finally, 9 mL of chitosan (CS) solution was taken, stirred under an ice-bath conditions, and β-glycerophosphate (β-GP) solution was added drop by drop to obtain a CS/β-GP hydrogel solution. Subsequently, while stirring, we added the SA solutions with different mass fractions according to the volume ratios of 2:1 and 1:1, and then added 0.1 mol/L acetic acid solution or 0.1 mol/L sodium hydroxide solution to adjust the pH of the solution to neutral. Statistics showed that when the volume ratio of CS/β-GP to 0.6 wt% SA was 2:1, the time from sol to gel state at 37 °C was the shortest and it was injectable.

We centrifuged the solution resuspended with BPNS at 10,000 rpm for 20 min and discarded the supernatant to obtain BPNS precipitation. We washed the precipitation with deionized water three times and then mixed it thoroughly with the above solution using ultrasonic treatment. This resulted in a mixed hydrogel solution containing 100 μg/mL of BPNS.

### 2.3. Characterization of BPNS and Hydrogels

By adjusting the ratio and concentration of CS/β-GP to SA, the gelation time of solutions with different proportions was detected at 37 °C using the inverted tube method. The hydrogel precursor solution was transferred into a 5 mL transparent glass vial, immersed in a thermostatic water bath maintained at 37 °C, and inverted every 30 s by 180° rotation. The gelation time was determined as the interval until complete loss of fluidity. And 1 mL syringes were used to test the injection condition of the hydrogel in the sol state.

The surface morphology and structure of BPNS and hydrogels were observed by scanning electron microscopy (SEM) and transmission electron microscopy (TEM). The steps are as follows: Take an appropriate amount of BPNS-containing solution and centrifuge at 10,000 rpm for 20 min. Discard the supernatant to obtain BPNS precipitate, followed by three washing cycles. Suspend the precipitate in a small amount of ethanol. After ultrasonic dispersion, drop the suspension onto a silicon wafer and allow it to air-dry naturally. Secure the sample on the specimen stage using conductive adhesive, then perform gold sputter-coating to enhance SEM imaging quality. For TEM observation: Mix the BPNS precipitate with pure water by ultrasonication, take approximately 20 μL of the sample, and drop it onto a specimen plate. Cover the droplet surface with a copper grid for 3 min before removal and air-drying. For hydrogel sample preparation: Rapidly freeze the hydrogel sample and sublimate moisture under vacuum. Carefully cut a section and adhere it to the SEM specimen stage using conductive adhesive. Perform gold sputter-coating to improve conductivity for optimal SEM observation. The size, shape, and surface characteristics of BPNS were observed via SEM and TEM. Meanwhile, the overall structure, pore characteristics (including shape, size, and distribution), and skeletal features of the hydrogel were examined using SEM. Additionally, the average pore size of hydrogel was calculated using ImageJ software (Version 1.54p).

In addition, the degradation of BPNS in water and hydrogels was detected by color change. BPNS was dispersed in water, its absorption spectra were measured, and photographs were taken on days 0, 2, 4, 6, and 8. The degradation rate was calculated based on a pre-established concentration–absorbance standard curve. Additionally, BPNS was embedded in hydrogels, and photographs were taken at predetermined time intervals. The degradation rate of the hydrogels was measured using the weight method. The hydrogels were freeze-dried, weighed as w0, and stored in PBS solution. The hydrogels were taken out on days 1, 3, 5, 7, 14, and 21 for measurement of the freeze-dried weight (wt), and the degradation rate was calculated using the formula $\frac{w0-\mathrm{wt}}{w0}\times 100\%$.

### 2.4. Cytotoxicity Assay

PC12 cells, a well-characterized neuroendocrine cell line, exhibit functional and phenotypic similarities to spinal cord neurons and are extensively utilized as an in vitro model for neurobiological studies.

The in vitro cytotoxicity of BPNS and CS/β-GP/SA hydrogels was detected using the CCK-8 assay. PC12 cells were seeded into 96-well plates at approximately 5000 cells per well and cultured overnight. The supernatant medium was then discarded and replaced with medium containing different concentrations of BPNS and CS/β-GP/SA hydrogel extracts. After culturing for 24 h, the medium was discarded, and the cells were washed twice with PBS. Fresh medium containing 100 µL with 10 µL of CCK-8 solution was added, and the cells were incubated in a light-protected environment at 37 °C and 5% CO_2_ for 2 h. An enzyme-labeled instrument was used to measure the OD value at 450 nm.

### 2.5. Cell Therapy

We seeded PC12 cells in a 96-well plate, ensuring about 5000 cells per well, and cultured overnight. We discarded the supernatant medium, and added medium containing H2O2 and different concentrations of BPNS to incubate for a certain period. Then, we assessed cell viability using the CCK-8 assay.

### 2.6. ROS Determination

The cellular ROS content was measured using a reactive oxygen species (ROS) assay kit (#S0033, Beyotime Institute of Biotechnology, Nantong, China). The DCFH-DA was diluted 1:1000 with serum-free medium to a final concentration of 10 μmol/L. After removing the culture medium from the six-well plate seeded with PC12 cells, 1.5 mL of the diluted DCFH-DA probe was added to each well. The plate was then incubated at 37 °C for 20 min in a CO_2_ incubator, with mixing every 5 min to ensure thorough probe–cell contact. The supernatant was subsequently aspirated, and the cells were washed three times with serum-free medium to remove excess DCFH-DA before fluorescence intensity was observed and recorded under a fluorescence microscope.

### 2.7. In Vivo Animal Models

The rats used in this study obtained ethical review approval in advance and authorization from the Animal Research Committee of Guangxi University (Approval No. GXU-2025-004). All male SD rats in this experiment were raised in an SPF environment, maintaining a constant temperature and humidity (humidity 60% ± 10%, temperature 24 °C ± 1 °C), with 12 h of light and 12 h of darkness daily, providing ample food and water. SD rats with an average body weight of 180 g were selected for each group (*n* = 9), weighed, and numbered and were divided into four groups. The Sham group only underwent laminectomy without spinal cord injury treatment. The SCI group underwent spinal cord transection and was injected with a PBS solution. The GEL group was injected with a hydrogel solution without BPNS after modeling, and the GEL-BPNS group was injected with a hydrogel solution containing BPNS (Figure S1).

The specific modeling method was as follows: The anesthetic dose was determined according to body weight and the rats were anesthetized with an intraperitoneal injection of 40 mg/kg pentobarbital sodium solution (Sigma-Aldrich, St. Louis, MO, USA). After anesthesia, the rats were fixed in a prone position on the experimental board, the back hair was shaved, and the skin was disinfected with iodophor swabs, making a 2 cm longitudinal incision centered on T9. The skin and fascia were cut open, the paravertebral muscles on both sides of the spinous process were bluntly separated, exposed using hemostats, and the vertebral spinous process was exposed. The spinous process and vertebral plate were removed to expose the spinal cord. A 2 mm section of the spinal cord was removed with ophthalmic scissors, and the muscle and skin were sutured. The rats were housed two per cage, placed in a well-ventilated area, with regular replacement of bedding, and provided with feed and water. The rat bladders were manually emptied twice a day, and 80,000 U of penicillin was injected intraperitoneally for three days after surgery.

On the 7th day after surgery, the rats were anesthetized with an intraperitoneal injection of 40 mg/kg pentobarbital sodium solution, followed by intracardiac perfusion with phosphate buffer solution. Then, 2 cm of spinal cord tissue above and below the center of the spinal cord injury was taken for molecular, biochemical, and immunohistochemical analysis. For histopathological analysis, the rats were sacrificed at 6 weeks after modeling, perfused with 4% paraformaldehyde, and the spinal cord samples were removed and stored in 4% paraformaldehyde at 4 °C.

### 2.8. BBB Score, Bladder, and Hindlimb Posture

The functional recovery of the rat’s hindlimbs was evaluated using the Basso, Beattie, and Bresnahan (BBB) locomotor score scale [40]. The BBB score ranges from 0 to 21. The rat to be tested was placed in an open field to become familiar with the environment, and then two independent observers who were unaware of the experimental groups assessed the BBB score according to the table. The BBB score and hindlimb posture were evaluated and recorded once a week for 6 weeks after surgery. The weight change of the rats was recorded after surgery to assess recovery. The recovery of urination was recorded when assisting with urination, and the bladder was weighed after the experiment.

### 2.9. Detection of Iron, Glutathione, and Malondialdehyde Content

The levels of iron, MDA, and GSH in rat spinal cord tissue were measured using an iron assay kit (A039-2-1; Nanjing Jiancheng Bioengineering Institute, Nanjing, China), an MDA assay kit (A003-1-2, Nanjing Jiancheng Bioengineering Institute), and a GSH detection kit (A006-2-1, Nanjing Jiancheng Bioengineering Institute). Spinal cord tissues were homogenized using a tissue homogenizer to obtain a 10% spinal cord homogenate. After centrifugation, the supernatant was collected, and samples were assayed according to the manufacturer’s instructions for the kit. Finally, absorbance values were measured using a microplate reader (Thermo Scientific Multiskan FC, Waltham, MA, USA).

### 2.10. Histological Analysis

Spinal cord samples were embedded in paraffin, cut into 4 μm thick sections, and de-waxed and hydrated with xylene and absolute alcohol. The sections were then stained with hematoxylin–eosin reagent and Nissl staining solution. Subsequent gradient dehydration, clearing, and mounting were performed, followed by observation under a light microscope. The TUNEL staining kit was used to detect neuronal apoptosis by incubating spinal cord tissue with terminal deoxynucleotidyl transferase, deoxyuridine triphosphate, and buffer at 37 °C for 1 h, followed by DAPI staining. The apoptotic cells and total cells were observed and counted under a fluorescence microscope, and the proportion was calculated to assess the degree of apoptosis.

### 2.11. Immunohistochemical Analysis

After antigen retrieval in EDTA buffer at pH 9.0, spinal cord sections were incubated with 3% hydrogen peroxide at room temperature in the dark for 25 min to block endogenous peroxidase activity. Subsequently, the sections were incubated with bovine serum albumin for 30 min, followed by addition of the primary antibody and incubation overnight at 4 °C. After washing the sections with PBS, the corresponding secondary antibody was added and incubated for 30 min at room temperature. Color development was performed using 3,3′-diaminobenzidine, and the sections were stained with hematoxylin for 3 min. After dehydration, the sections were mounted with neutral resin and imaged using a microscope.

### 2.12. Western Blotting

Cells and spinal cord proteins were subjected to Western blot analysis using the following antibodies: NRF2 (1:5000, Proteintech, 16396-1-AP, Wuhan, China), HO-1 (1:5000, Proteintech, 10701-1-AP, Wuhan, China), NQO1 (1:4000, Proteintech, 11451-1-AP, Wuhan, China), ACSL4 (1:6000, Proteintech, 22401-1-AP, Wuhan, China), xCT (1:3000, Immunoway, YM8243, Suzhou, China), GPX4 (1:2000, Proteintech, 30388-1-AP, Wuhan, China), BAX (1:5000, Proteintech, 50599-2-Ig, Wuhan, China), BCL-2 (1:2000, Proteintech, 26593-1-AP, Wuhan, China), Caspase-3 (1:2000, Proteintech, 19677-1-AP, Wuhan, China), Beta Actin (1:5000, Proteintech, 20536-1-AP, Wuhan, China). Protein bands were visualized using the Western ECL Detection Kit (Beyotime, P0018FM, Shanghai, China) and detected with a chemiluminescent imaging system (Beijing SinSage Technology Co., Ltd., Beijing, China).

### 2.13. Transcriptomic Analysis

The spinal cord tissue was quick-frozen in liquid nitrogen, then ground into a fine powder and added to an RNAase-free centrifuge tube containing Trizol reagent for RNA isolation, detection, high-throughput sequencing, and data analysis to identify differentially expressed genes. Functional annotation of the differentially expressed genes was performed using the Gene Ontology (GO) database and the Kyoto Encyclopedia of Genes and Genomes (KEGG) database to determine the functional categories and biological pathways.

### 2.14. Statistical Analysis

All data analyses were conducted using GraphPad Prism version 9.0. All tests were repeated three times or more. One-way analysis of variance was used for multiple-group comparisons and t-tests were used for two-group comparisons. The results are shown as the mean ± standard deviation (SD). A value of *p* < 0.05 indicates significance.

## 3. Results

### 3.1. Synthesis and Detection of the Composite

**Figure 1:**
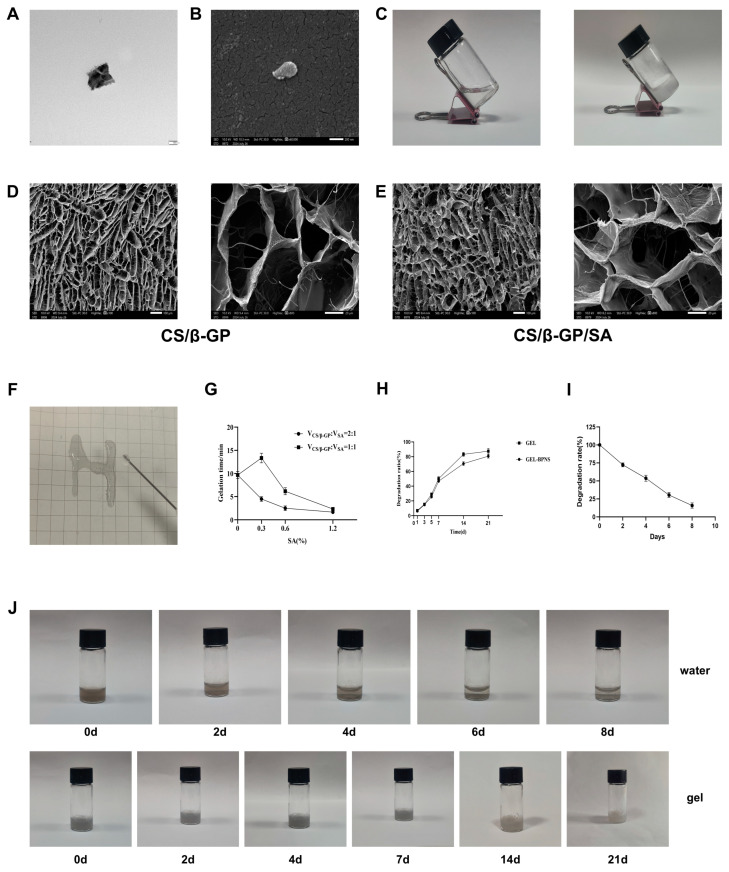
Characterization of BPNS and hydrogels. (**A**) Scanning electron microscopy (SEM) image of BP NS, scale bar = 100 nm. (**B**) Transmission electron microscopy (TEM) image, scale bar = 200 nm. (**C**) Hydrogel transition from solution to gel at 37 °C. (**D**,**E**) SEM images of hydrogel and BPNS-loaded hydrogel, with scale bars of 20 μm and 100 μm, respectively. (**F**) Schematic illustration of the injectability of the hydrogel at room temperature. (**G**) Time required for the hydrogel to transition from solution to gel after adding SA at different concentrations and proportions. (**H**) Degradation rate of the hydrogel. (**I**) Degradation rate of the BPNS. (**J**) Schematic illustration of the degradation of BPNS in aqueous solution and hydrogel.

The morphology of BPNS was observed using scanning electron microscopy (SEM) and transmission electron microscopy (TEM). As shown in Figure 1A,B, BPNS had a lamellar structure with a diameter of about 200 nm. The inclined glass sample tube displayed the transition of the hydrogel from a liquid to a gel state, indicating that the hydrogel had temperature-sensitive properties (Figure 1C) [41]. SEM analysis revealed that the hydrogel exhibited an irregular yet uniformly porous architecture. The average pore size of the CS/β-GP hydrogel was 60 μm, while that of the CS/β-GP/SA hydrogel was 66 μm. The pores of the hydrogel with added SA did not show significant changes, but the pore walls became thicker, suggesting enhanced mechanical structure of the hydrogel (Figure 1D,E). This demonstrated that the crosslinking of β-GP with chitosan formed a porous structure. The syringe injection showed that the synthesized hydrogel was injectable (Figure 1F). As shown in Figure 1F, statistical findings revealed that at a ratio of CS/β-GP to 0.6 wt% SA of 2:1, the time from sol state/to gel state at 37 °C was the shortest and it possessed injectability. At the same time, there was no significant difference in the degradation rate between the GEL and GEL-BPNS, both having a stable degradation rate, which was conducive to the release of BPNS, allowing it to be gradually removed in the body without producing toxic effects (Figure 1G,H). UV–Vis absorption spectroscopy was used to investigate the degradation of BPNS. The nanosheets degraded by 84.2% within 8 days (Figure 1I). As shown in Figure 1J, BPNS dispersed in the aqueous solution was basically degraded after 8 days, while in the hydrogel it could last for 21 days, indicating that using hydrogel to load BPNS could significantly reduce its degradation rate. These results indicated that we had successfully synthesized an injectable temperature-sensitive hydrogel that could rapidly gelate, effectively slowing down the release of BPNS.

### 3.2. Composite Promotes Spinal Cord Injury Rat Functional Recovery

To study the effect of BPNS on behavioral recovery after spinal cord injury in rats, a hydrogel containing BPNS was added to the rats after spinal cord injury, while another group was only given a hydrogel without BPNS. A group with an equal amount of PBS was used as the modeling group, and a sham operation group had only the rat’s vertebral plate removed without spinal cord injury treatment.

**Figure 2:**
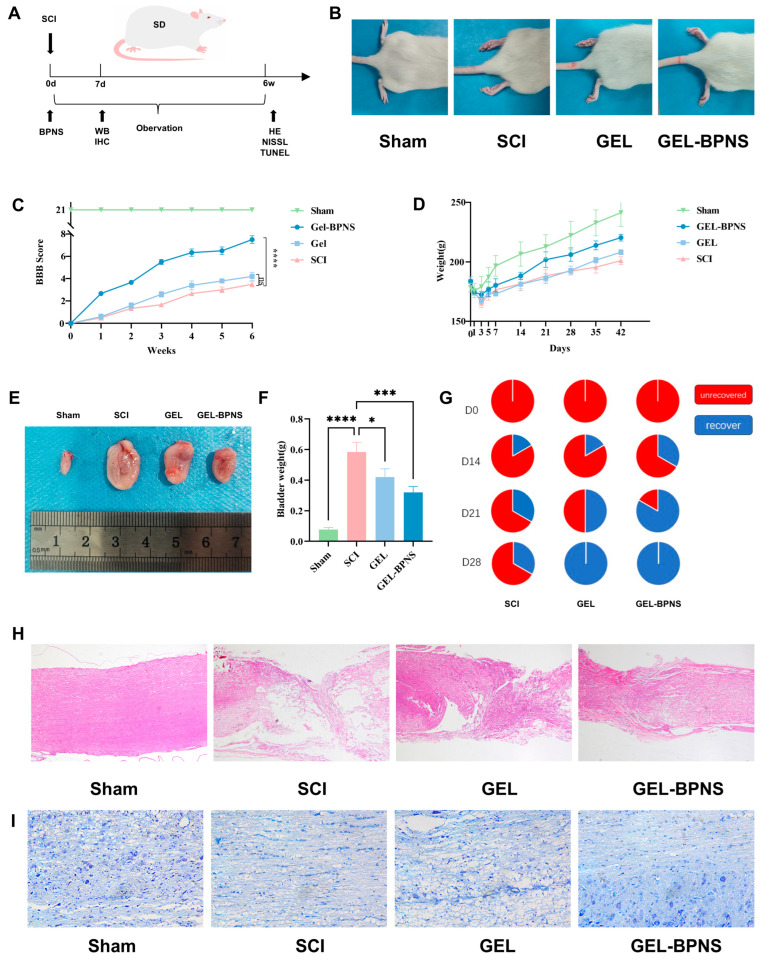
Functional recovery of spinal cord injured rats. (**A**) Experimental procedure. (**B**) Hindlimb posture diagrams of rats in each group (*n* = 6). (**C**) BBB score results of rats in each group from 0 to 6 weeks after SCI (*n* = 6). (**D**) Trends of body weight changes in rats of each group from 0 to 6 weeks after SCI (*n* = 6). (**E**) Comparison chart of rat bladder size. (**F**) Statistical comparison of bladder weight in rats of each group (*n* = 6). (**G**) Urinary recovery of rats in each group from 0 to 28 days (*n* = 6). (**H**,**I**) HE (microscope’s magnification: 40×)and Nissl (microscope’s magnification: 200×) staining images of rats in each group. * *p* < 0.05, *** *p* < 0.001, **** *p* < 0.0001, ns: no significant difference.

The postoperative body weight, urination, and motor recovery of the rats were monitored as shown in Figure 2A. Figure 2B shows the hindlimb morphology of the rats in each group during movement at 6 weeks, with at least one hindlimb in the GEL-BPNS group being able to touch the ground with the palm of the paw without weight-bearing. Figure 2C shows that according to the BBB score results from day 0 to 6 weeks after spinal cord injury [40], the scores of all groups except the sham group dropped to 0 and then began to rise over time. The BP group’s score at the same time was significantly higher than that of the GEL and SCI groups, indicating that the GEL-BPNS group had a more favorable influence on the recovery of motor function in rats. In the first three days, due to surgical modeling, the body weight of the rats decreased rapidly, except for the laminectomy group, and then began to rise. Compared with the modeling group and the hydrogel group, the drug administration group recovered the fastest, suggesting that the treatment group had the fastest recovery speed (Figure 2D). The rat bladder images taken 6 weeks later are shown in Figure 2E; the bladder volume of the SCI group was significantly increased, the GEL group slightly smaller than the SCI group, and the GEL-BPNS group significantly smaller than the SCI group, proving that both GEL and GEL-BPNS could reduce pathological hypertrophy of the rat bladder. Based on the recovery of urination reflex and the weight of the bladder after 6 W, GEL-BPNS had the effect of promoting urination recovery and reducing pathological bladder hypertrophy (Figure 2F,G). Figure 2H,I show that after 6 W, the spinal cord was taken for HE and Nissl staining, and the results show that both the GEL and GEL-BP groups could reduce the cavity after spinal cord injury, increase the number of nerve cells, and reduce tissue inflammatory infiltration, with the GEL-BPNS group showing more significant effects. From the above results, it can be seen that GEL-BPNS could effectively improve motor and urination dysfunction in rats with spinal cord injury, reduce tissue damage, and increase the number of neurons.

### 3.3. Compound Cytotoxicity and Antioxidant Properties

**Figure 3:**
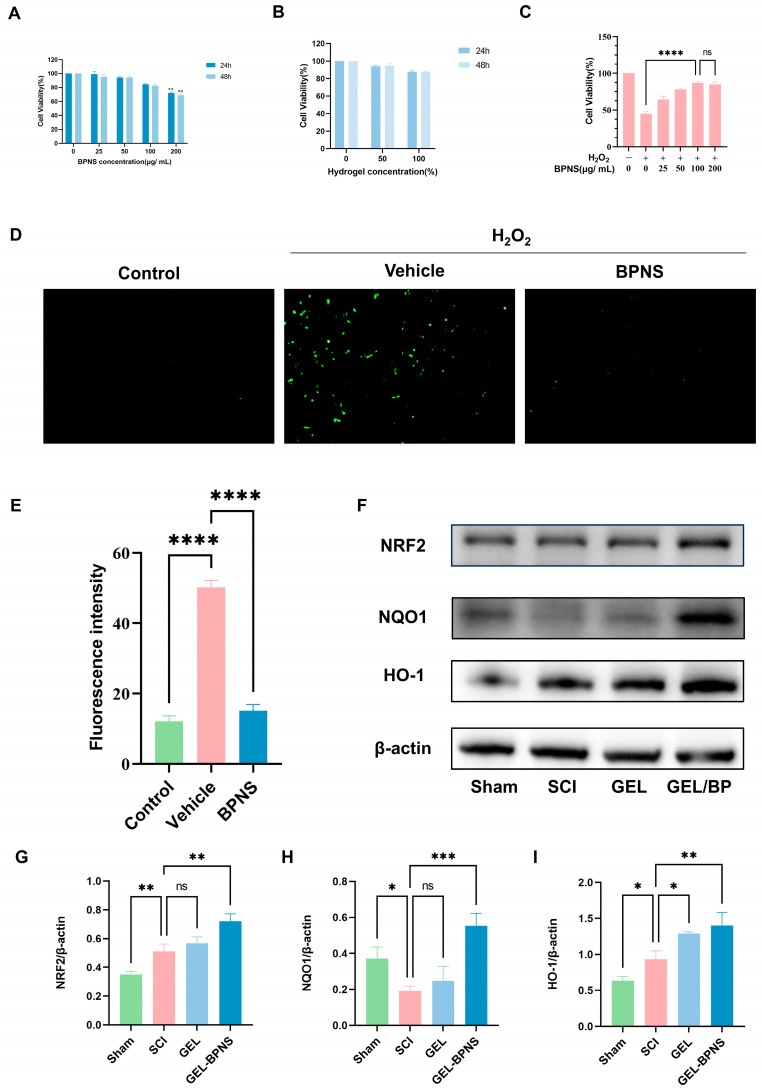
BPNS and hydrogel in vitro compatibility and antioxidant activity. (**A**) Cytotoxicity of different concentrations of BPNS on PC12 cells. (**B**) Cytotoxicity of different concentrations of hydrogel extracts on PC12 cells. (**C**) Protective effect of BPNS on PC12 cells treated with H_2_O_2_. (**D**) ROS fluorescence intensity images of PC12 cells. 200×. Scale bar = 100 μm. (**E**) Quantitative analysis of ROS intensity. (**F**) Western blotting showing the expression of NRF2, NQO1, and HO-1. (**G**) Quantitative analysis of NRF2 protein expression (*n* = 3). (**H**) Quantitative analysis of NQO1 protein expression (*n* = 3). (**I**) Quantitative analysis of HO-1 protein expression (*n* = 3). * *p* < 0.05, ** *p* < 0.01, *** *p* < 0.001, **** *p* < 0.0001, ns: no significant difference.

To evaluate the cytotoxic effects of BPNS on cells, different concentrations of BPNS were resuspended in culture medium and co-cultured with PC12 cells for 24 and 48 h to assess cell viability. As shown in Figure 3A, BPNS at concentrations ranging from 0 to 100 μg/mL exhibited no significant cytotoxicity after co-culture with PC12 cells, with cell viability remaining above 80%. Additionally, the results from co-culturing cells with culture medium extracts of 50% and 100% hydrogels demonstrated the negligible cytotoxicity of the hydrogels (Figure 3B). Different concentrations of BPNS were added to cells treated with H_2_O_2_, and the results showed that BPNS could increase cell survival rates (Figure 3C). To detect the protective effect of BPNS on cells treated with H_2_O_2_, a DCFH-DA probe was added to cells treated with or without nanosheets in the presence of H_2_O_2_, and fluorescence intensity was observed and counted using a fluorescence microscope. BPNS significantly reduced the intracellular ROS content (Figure 3D,E). To further verify the protective mechanism of BPNS against oxidative stress, Western blotting was used to detect related pathways (Figure 3F). The results shown in Figure 3G–I indicated that compared to the Sham group, the SCI group increased the expression of NRF2 and HO-1 while decreasing the expression of NQO1, suggesting that BPNS reduced ROS levels and H_2_O_2_-induced oxidative stress through NRF2 pathway regulation. There was no significant difference between the GEL group and the SCI group, indicating that GEL had a minor impact on the expression level of pathway proteins. The GEL-BPNS group significantly increased the expression levels of NRF2, HO-1, and NQO1. The above results demonstrated that the composite was non-cytotoxic and could reduce ROS production through the Nrf2 pathway and exert antioxidant effects.

### 3.4. Transcriptomic Analysis

**Figure 4:**
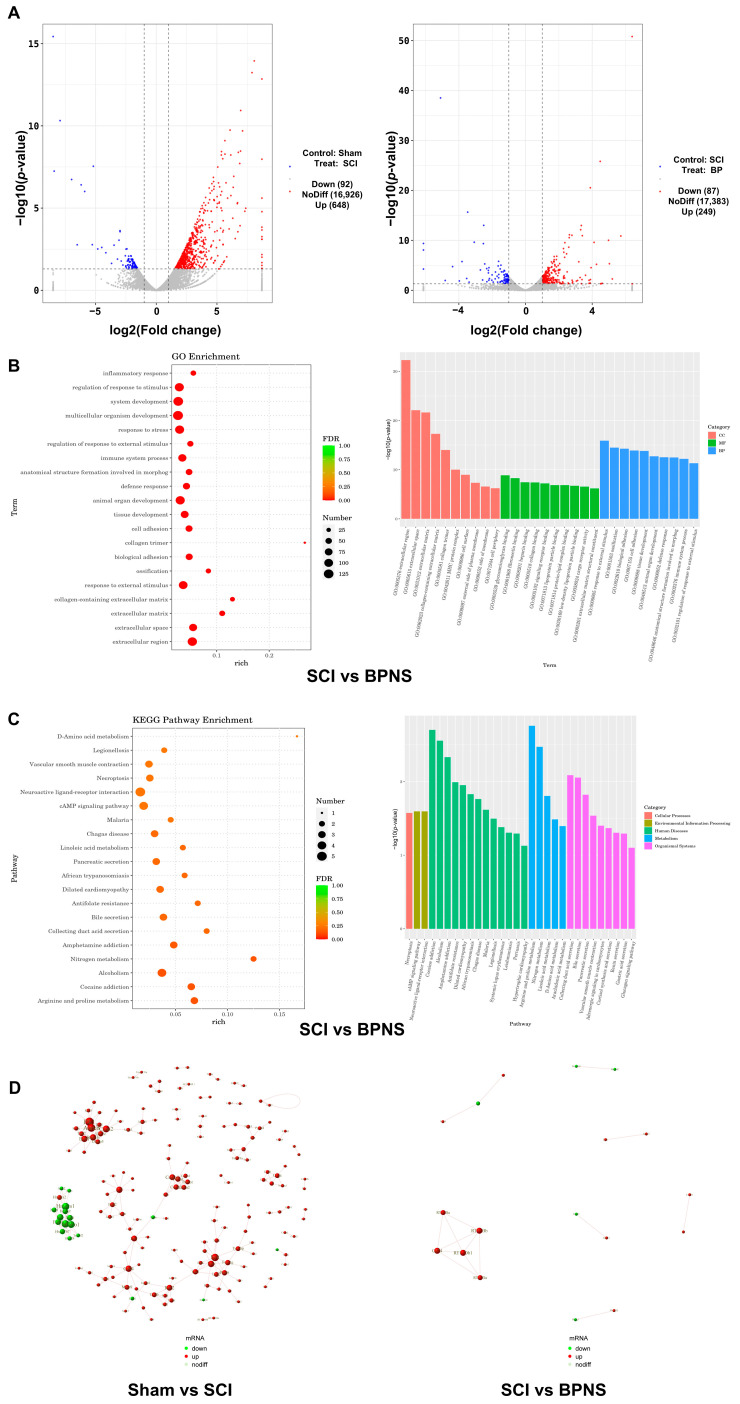
Transcriptomic analysis between the Sham group, SCI group, and BP group. (**A**) Volcano plots of differentially expressed mRNA between the Sham and SCI groups, and between the SCI and BP groups. The vertical dotted lines mark the cut-off values for log2(Fold Change) that distinguish between up-regulated and down-regulated genes. The horizontal dotted line indicate the significance level for the −log10(*p*-value), beyond which the results are considered statistically significant. (**B**) GO enrichment analysis of differentially expressed mRNA between the SCI and BP groups. (**C**) KEGG pathway enrichment analysis of differentially expressed mRNA between the SCI and BP groups. (**D**) Protein–protein interaction networks of differentially expressed mRNA between the Sham and SCI groups, and between the SCI and BP groups, indicating the dynamic changes in protein interactions and the relationship between these changes and physiological or pathological processes.

To further the study, we conducted transcriptomic analysis to investigate the differences in spinal cord tissue mRNAs among the Sham group, SCI group, and GEL-BPNS group. As shown in Figure 4A, compared to the Sham group, the SCI group had 648 upregulated mRNAs and 92 downregulated mRNAs; compared to the SCI group, the GEL-BPNS group had 249 upregulated mRNAs and 87 downregulated mRNAs.

Gene Ontology (GO) enrichment analysis was categorized into three aspects: cellular component (CC), molecular function (MF), and biological process (BP). The top ten GO term entries in each category were selected for display. The results showed relevance to terms such as 0009605—response to external stimulus, 0032101—regulation of response to external stimulus, 0005518—collagen binding, 0005539—glycosaminoglycan binding, 0006952—defense response, etc. (Figure 4B). Kyoto Encyclopedia of Genes and Genomes (KEGG) pathway analysis revealed enrichment in Necroptosis, cAMP Signaling Pathway, Nitrogen Metabolism, Arachidonic Acid Metabolism, etc. (Figure 4C). These enrichment analysis results were related to cellular stress responses, immune regulation, and metabolic disorders. The PPI results in Figure 4D showed the dynamic changes in protein interactions and the relationship between these changes and physiological or pathological processes.

### 3.5. The Composite Inhibits Ferroptosis After SCI by Activating the xCT/GSH/GPX4 Signaling Pathway

**Figure 5:**
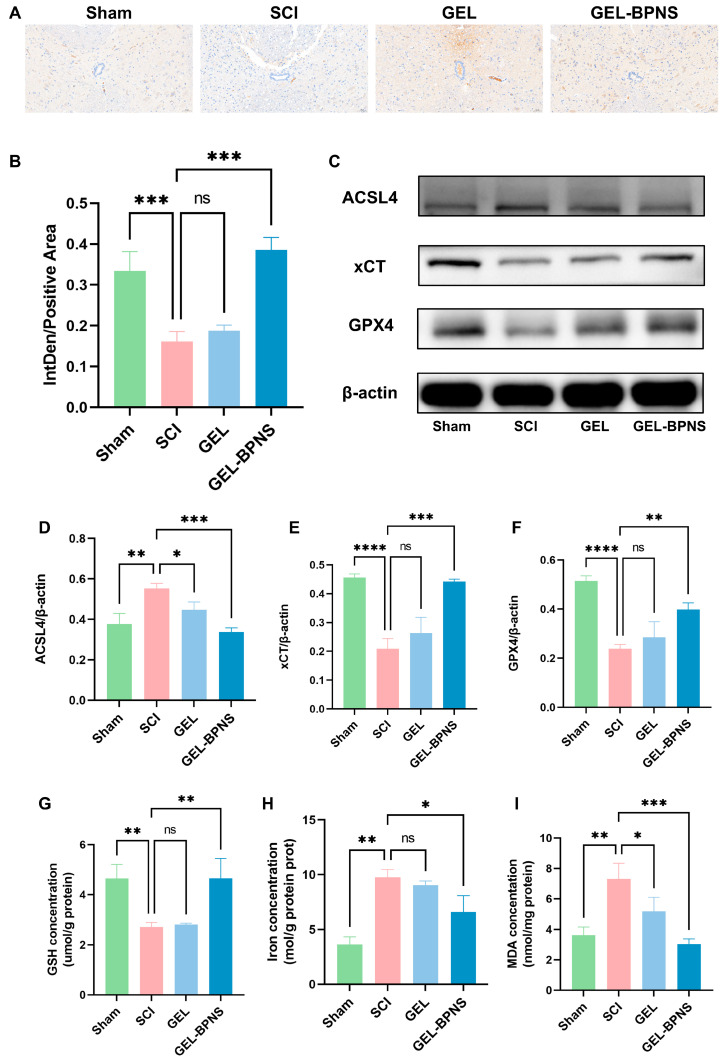
Expression of ferroptosis-related proteins in each group. (**A**) Immunohistochemical illustration of GPX4 in each group. 200×. Scale bar = 50 μm. (**B**) Quantitative statistics of GPX4 immunohistochemistry in each group (*n* = 5). (**C**) Western blotting results of ACSL4, xCT, and GPX4. (**D**) Quantitative analysis of ACSL4 protein expression (*n* = 3). (**E**) Quantitative analysis of GPX4 protein expression (*n* = 3). (**F**) Quantitative analysis of xCT protein expression (*n* = 3). (**G**) Statistics of GSH content in spinal cord tissue of each group (*n* = 3). (**H**) Statistics of iron content in spinal cord tissue of each group (*n* = 3). (**I**) Statistics of MDA content in spinal cord tissue of each group (*n* = 3). * *p* < 0.05, ** *p* < 0.01, *** *p* < 0.001, **** *p* < 0.0001, ns: no significant difference.

The spinal cord tissue of rats was examined at 7 days after modeling. Figure 5A showed the GPX4 immunohistochemical image. As shown in Figure 5B, the immunohistochemical results revealed that the percentage of GPX4 positive area in the SCI group was lower than that in the Sham group, while the GEL-BPNS group was significantly higher than the SCI group. Western blotting results showed (Figure 5C) that after spinal cord injury, compared with the Sham group, the expression levels of GPX4 and xCT in the SCI group significantly decreased, and the expression level of ferroptosis-positive product ACSL4 significantly increased. The GEL-BPNS group reversed these trends (Figure 5D–F). As shown in Figure 5G–I, the MDA and iron contents in the GEL-BPNS group were significantly lower than those in the SCI group, while the GSH content was significantly higher than that in the SCI group. These results indicated that GEL-BPNS could activate the GPX4 pathway in vivo, inhibit ferroptosis, and exert neuroprotective effects.

### 3.6. Composite Inhibits Apoptosis After SCI

**Figure 6:**
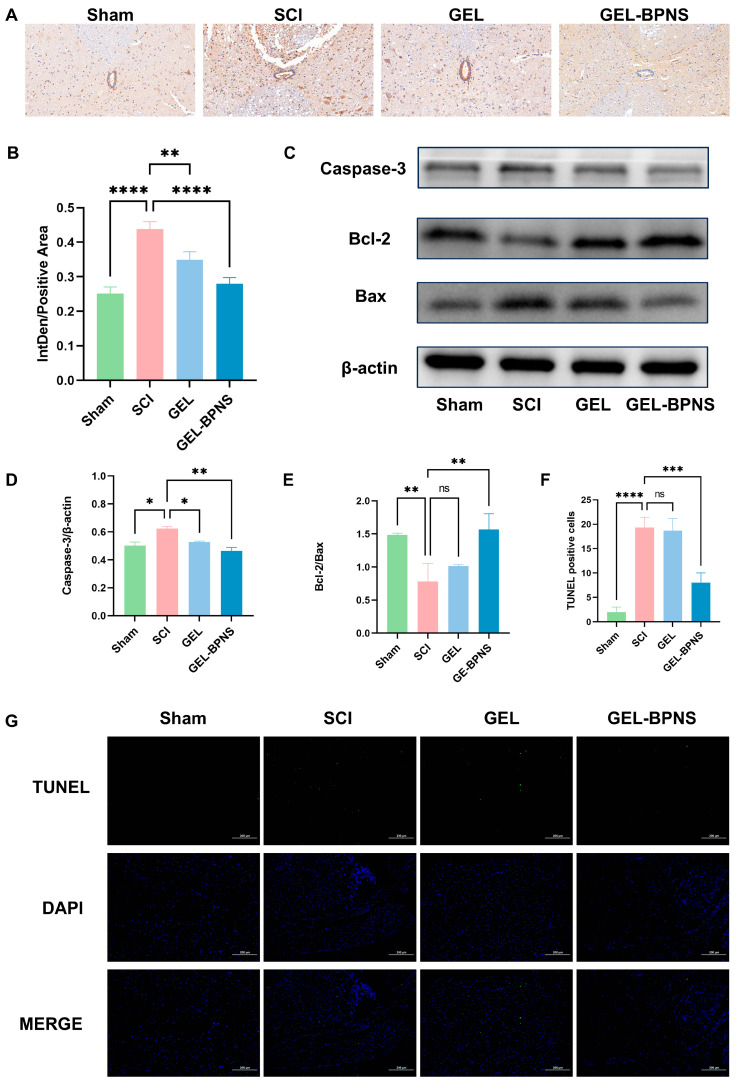
The effect of GEL-BPNS on apoptosis in rat spinal cord. (**A**) Immunohistochemical illustrations of Caspase-3 in each group. 200×. Scale bar = 50 μm. (**B**) Quantitative statistics of Caspase-3 immunohistochemistry in each group (*n* = 5). (**C**) Western blotting results of Caspase-3, Bcl-2, and Bax. (**D**) Quantitative +analysis of Caspase-3 protein expression (*n* = 3). (**E**) Quantitative analysis of the ratio of Bcl-2 and Bax protein expression (*n* = 3). (**F**) Statistical analysis of TUNEL-positive cells in each group. 200×. Scale bar = 200 μm (*n* = 6). (**G**) TUNEL staining illustrations in each group. * *p* < 0.05, ** *p* < 0.01, *** *p* < 0.001, **** *p* < 0.0001, ns: no significant difference.

To further investigate the effect of GEL-BPNSN on apoptosis in rat spinal cord neurons, immunohistochemical results showed that GEL-BPNSN could reduce the expression of Caspase-3 in the rat spinal cord after injury (Figure 6A,B). As shown in Figure 6C, the expression of apoptosis-related proteins was detected by Western blotting. The results indicated that after spinal cord injury, the expression of pro-apoptotic protein Caspase-3 increased, and the Bcl-2/Bax ratio decreased. However, the addition of GEL-BPNS significantly reduced the expression of Caspase-3 and increased the Bcl-2/Bax ratio (Figure 6D,E). TUNEL staining results showed a large number of positive apoptotic cells in the SCI group, while the number of positive apoptotic cells in the GEL-BPNS group was significantly reduced (Figure 6F,G). In summary, GEL-BPNS could inhibit apoptosis after SCI.

## 4. Discussion

In spinal cord injury, oxidative stress and ferroptosis are important pathways leading to neuronal death [8,42]. BPNS has an excellent ROS scavenging ability, which can effectively reduce oxidative stress responses [24,27]. However, BPNS is characterized by being highly prone to degradation [43,44]. In view of this, this study aims to employ a loading system that can slow down the degradation rate of BPNS to enhance its effectiveness and investigate its specific mechanisms of action in rat spinal cord injury.

According to the literature research, CS can form a temperature-sensitive hydrogel under the crosslinking action of β-GP, which can be used as a drug delivery carrier [30]. CS/β-GP hydrogel carrying nerve growth factor for rat spinal cord injury can continuously release nerve growth factor at the injury site, showing significant therapeutic effects [45]. The interaction between SA and CS can change the properties of CS/β-GP hydrogel [33]. Therefore, we explored the properties of hydrogels obtained by mixing SA solutions with different concentrations and volume ratios with CS/β-GP solutions, and selected a hydrogel with significantly shortened gelation time that was injectable. Compared to conventional thermosensitive hydrogels requiring 10–20 min for gelation at 37 °C [46], we successfully developed an injectable temperature-sensitive hydrogel with a significantly shortened gelation time (≤2.5 min at 37 °C).

Subsequently, we used electron microscopy to examine the basic structure of BPNS and the hydrogel. The hydrogel exhibits a three-dimensional porous network structure with irregular pore shapes and relatively uniform distribution. This porosity enables efficient diffusion of nutrients (e.g., glucose, growth factors) and removal of metabolic waste, supporting cell viability within the hydrogel [47]. For the BPNS-loaded system, the porous architecture also facilitates controlled release of nanosheets, ensuring sustained delivery of antioxidants to the injury site. And we observed the degradation of the hydrogel and BPNS in different media. These results demonstrated the obtaining of an effective drug delivery system that could quickly form a stable gel structure at the injury site, thereby achieving precise and sustained drug release.

HE and Nissl staining results indicated that BPNS successfully reduced tissue necrosis, maintained spinal cord tissue integrity, and reduced neuronal damage, creating favorable conditions for the recovery of neurological function. The increase in the number of Nissl bodies suggests that the metabolic activity and function of neurons may have been enhanced, further confirming that BPNS has a certain protective effect on neurons. The recovery of motor function indicates that GEL-BPNS plays a role in aspects such as neural signal conduction. Spinal cord injury is often accompanied by urinary system dysfunction [48], and the recovery of urination suggests that GEL-BPNS has potential improvement effects on complications caused by spinal cord injury.

By directly applying transcriptomic analysis to study the differences in mRNAs between different groups, it is possible to quantitatively analyze the level of gene expression at the whole-genome level [4,49]. By comparing the expression status of mRNA in different groups, it can be systematically found that after spinal cord injury, GEL-BPNS intervention mainly plays a role in affecting lipid metabolism-related processes. In the oxidative stress process caused by spinal cord injury, the accumulation of ROS is a key reason for cell death [50]. Oxidative stress is a stress response produced by cells after being stimulated exogenously, resulting in the generation of ROS and other oxidative substances, which in turn triggers the ferroptosis process [51]. GEL-BPNS may control the synthesis of glutathione (GSH) by regulating the activity of cysteine/glutamate reverse transporter (xCT), thus inhibiting the process of ferroptosis. Studies have shown that eliminating ROS can inhibit lipid peroxidation, thereby reducing ferroptosis. The experimental results show that GEL-BPNS reduced the content of ROS in PC12 cells treated with H₂O₂, and the increase in the survival rate of PC12 cells also verified the result of GEL-BPNS reducing cell death. The Nrf2 pathway occupies a central position in the cellular antioxidant system, and the activation of the Nrf2/HO-1/NQO1 pathway can reduce oxidative stress caused by the accumulation of ROS [52,53,54]. Our research found that the activation of this pathway by GEL-BPNS enhanced the endogenous antioxidant capacity of cells and reduced ROS content, thereby reducing cell damage. The xCT/GSH/GPX4 signaling pathway is the main protective system against ferroptosis [55]. Our research results show that in the tissues of rats with spinal cord injury, with the increase in iron and ROS content, the expression of the key ferroptosis gene GPX4 was inhibited, indicating that ferroptosis plays an important role in the process of spinal cord injury. GEL-BPNS reduces the occurrence of ferroptosis by reducing ROS content at the root, and by activating the xCT/GSH/GPX4 pathway, it plays a role in inhibiting ferroptosis. The decrease in lipid peroxidation product MDA, the unsaturated fatty acid synthesis enzyme ACSL4, and the iron ion content also confirm this conclusion.

Apoptosis is a form of programmed cell death, and Caspase-3 is a key protease in the execution phase of cell apoptosis [13]. A decrease in its expression level indicates the inhibition of the cell apoptosis program [56]. Apoptotic neurons in spinal cord tissue can also be observed by Nissl staining [57]. Normal neuron cell bodies are full, with evenly distributed, deeply stained Nissl bodies, showing a typical blocky or granular pattern; apoptotic neuron cell bodies shrink, the number of Nissl bodies drastically decreases, and they are almost non-existent in severe cases, with cell boundaries being blurred. The results of this study showed that GEL-BPNS could reduce the expression level of Caspase-3 and increase the content of Bcl-2/Bax, strongly demonstrating the inhibitory effect of GEL-BPNS on spinal neuron apoptosis at the molecular level. TUNEL staining intuitively reflects the outcome of neuron apoptosis and is an important means to evaluate the therapeutic effect at the cellular apoptosis level [58]. TUNEL staining specifically labels fragmented DNA in apoptotic cells, thus clearly showing a reduction in spinal neuron apoptosis in the GEL-BPNS group at the histological level. In addition, the results of Nissl staining further validated this. These results proved that GEL-BPNS can effectively inhibit neuron apoptosis and exert neuroprotective effects.

The observed correlation between functional recovery (e.g., improved BBB scores) and biochemical improvements (e.g., reduced lipid peroxidation and apoptosis) underscores the multifactorial therapeutic mechanism of GEL-BPNS. Specifically, the hydrogel’s sustained release of BPNS ensures continuous ROS scavenging, which breaks the vicious cycle of oxidative stress–ferroptosis crosstalk. Moreover, the downregulation of pro-apoptotic Bax and the upregulation of anti-apoptotic Bcl-2 align with reduced caspase-3 activity, collectively contributing to the preservation of spinal cord tissue integrity and functional axons. Such spatiotemporal coordination between biochemical modulation and physiological outcomes highlights the translational advantage of our combinatorial strategy over monotherapeutic approaches [59].

Our research provided a treatment method for rat spinal cord injury using injectable thermosensitive hydrogels loaded with BPNS. The hydrogel effectively slowed down the degradation rate of BPNS, and the study reflected the therapeutic effects of GEL-BPNS, which could inhibit oxidative stress, ferroptosis, and neuronal apoptosis, showing significant neuronal protective effects. However, the current research still has certain limitations. Although in vitro cytotoxicity assays demonstrated a good biocompatibility of 0–100 μg/mL BPNS and the hydrogel within 48 h (Figure 3A,B), long-term toxicity—such as the sustained accumulation of the degradation product phosphate or the potential effects of hydrogel degradation intermediates—requires further validation through in vivo chronic toxicity studies. Systemic evaluation of long-term safety and side effects in large animal models remains essential for future work. In addition to reducing neuronal damage, the role of the hydrogel system loaded with BPNS in providing a supportive microenvironment for cell adhesion in terms of neuronal regeneration and promoting angiogenesis has not been further explored. The specific details of axon regeneration and the repair of synaptic connections between neurons have not been deeply investigated, so the comprehensive protective and repair effects of BPNS on neurons cannot be fully determined. The evaluation of urinary system function focuses only on the recovery of urination, and no in-depth detection has been conducted on other important aspects such as the composition of urine and the recovery of neural regulatory mechanisms of the urinary system, making it impossible to fully judge its impact on the overall function of the urinary system. At the same time, this study provides a new idea for the treatment of spinal cord injury, such as developing more intelligent hydrogels that can precisely release BPNS based on changes in the environment of the spinal cord injury site, such as changes in temperature, pH, enzyme concentration, etc. Ligands targeting the spinal cord injury site can also be added to the surface of BPNS to enhance therapeutic specificity. In addition, it can be combined with traditional treatment methods, for example, removing foreign bodies and necrotic tissue from the injury site after surgery to facilitate better adhesion of the hydrogel. In summary, GEL-BPNS has good application prospects in spinal cord injury and other nervous system diseases.

## 5. Conclusions

We successfully prepared an injectable temperature-sensitive hydrogel with rapid gelation, robust structural integrity, and the ability to efficiently load BPNS for in situ delivery. Our study showed that BPNS can alleviate cell death caused by H_2_O_2_-induced ROS elevation in vitro. In a rat SCI model, the hydrogel-loaded BPNS increased the expression level of GPX4, reduced the level of lipid peroxidation, inhibited ferroptosis, activated the caspase-3 signaling pathway, suppressed apoptosis, and reduced neuronal damage. Conclusively, this study paves the way for translational research by demonstrating the feasibility of a dual-function BPNS–hydrogel system in a rat SCI model. Subsequent steps will involve chronic toxicity studies in large-animal models to evaluate biocompatibility and long-term safety, focusing on BPNS metabolite accumulation and sustained immune responses over extended periods.

## Data Availability

The data presented are available from the corresponding author upon request.

## Supplementary Materials

The following supporting information can be downloaded at: https://www.mdpi.com/article/10.3390/pharmaceutics17050573/s1, Figure S1: Surgical procedure of spinal cord transection and implantation of the GEL-BPNS.
