# Supplementary material for: Synergistic Antioxidant and Anti-Ferroptosis Therapy via BPNS-Encapsulated Thermoresponsive Chitosan Hydrogel for Spinal Cord Injury Regeneration
Source: Pharmaceutics. 2025 Apr 26;17(5):573. doi: 10.3390/pharmaceutics17050573 (PMC12114966; doi:10.3390/pharmaceutics17050573)
Supplement: Supplementary file 1 [file pharmaceutics-17-00573-s001.zip › pharmaceutics-3559705-supplementary.pdf]

Supproting information

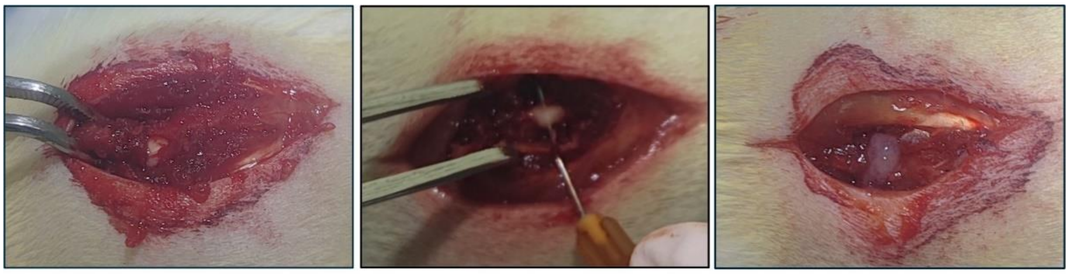

Spinal cord exposure **Spinal cord transection** **Hydrogel implantation**

Figure S1. Surgical procedure of spinal cord transection and implantation of the GEL-BPNS.
